# Minimally invasive surgery for ovarian endometriosis as a mean of improving fertility: Cystectomy vs. CO2 fiber laser ablation what do we know so far?

**DOI:** 10.3389/fsurg.2023.1147877

**Published:** 2023-03-27

**Authors:** Massimo Candiani, Jessica Ottolina, Noemi Salmeri, Sara D’Alessandro, Iacopo Tandoi, Ludovica Bartiromo, Matteo Schimberni, Stefano Ferrari, Roberta Villanacci

**Affiliations:** Obstetrics and Gynecology Department, San Raffaele Scientific Institute, Milan, Italy

**Keywords:** infertility, minimally invasive surgery, endometriosis, cystectomy, CO2 laser

## Abstract

Minimally invasive surgery emerged in the 1980s as a safe and effective technique which requires smaller incisions and, usually, a shorter hospital stay compared to traditional surgery. Since then, minimally invasive surgery has expanded in many surgical specialties. One of its newest application in gynecology stands in the infertility management of young women with unexplained infertility or suspected endometriosis. In these cases, laparoscopy allows to diagnose and treat the disease aiming to increase at best the chances of spontaneous pregnancy or trough assisted reproductive technology. Nowadays, minimally invasive surgical approach of ovarian endometriosis consists of either laparoscopic cystectomy or ablative techniques such as laparoscopic CO2 fiber laser vaporization. Although cystectomy represents the gold standard according to the latest Cochrane review, some endometriosis experts are worried about its detrimental effect on healthy ovarian parenchyma and suggest preferring a less aggressive approach such as CO2 fiber laser vaporization. The aim of this review is to give an overview of the available evidences about the impact of the two surgical procedures on ovarian reserve markers and pregnancy outcome.

## 1. Introduction

Minimally invasive surgery has made incredible progress in many specialties during the last decades. Particularly, surgery for ovarian disease has deeply changed from twenty years ago thanks to the introduction of newer and newer surgical techniques with the aim of preserving and improving fertility. This evolution has allowed to treat an increasing number of cases broadening the application of ovarian surgery in reproductive field (1). Undoubtedly, it is the responsibility of both the gynecological surgeon and the assisted reproductive technology specialists to evaluate the infertile couple so as to perform surgery effectively in properly selected patients.

Laparoscopy should be considered as a first-step approach in patients with symptoms of endometriosis (dysmenorrhea, chronic pelvic pain) and related infertility, in patients with a history of pelvic inflammatory disease, or previous ectopic pregnancy (2, 3). Special care must be put in cases of previous surgery. The primary aim of every endometriosis specialists should be to limit surgeries especially in women with ovarian lesions. Indeed, excisional surgery for recurrent endometriomas appears to be associated with histologic evidence of higher loss of heathy ovarian tissue if compared with primary surgery and may be more harmful to the ovarian reserve (4). When repeated surgery is combined with an advanced age, known to impact itself on ovarian reserve, the detrimental effect might be even worse. There may be also a place for laparoscopy for young women with a long duration (>3 years) of infertility but no recognized abnormalities (“prolonged unexplained infertility”) and for women with unexplained infertility and failed assisted reproductive technologies (ART) (2). Moreover, minimally invasive surgery plays an important role in the treatment/is part of the treatment of endometriosis, cesarean scar defects, tubal disease, congenital uterine malformations and uterine cavity abnormalities (5).

## 2. Minimally invasive surgery for endometriosis-associated infertility

Laparoscopy could be offered as a treatment option for endometriosis-associated infertility in stage I–II endometriosis as it improves the rate of ongoing pregnancy according to Revised American Society for Reproductive Medicine classification (rASRM) (3, 6). Despite this evidence, performing diagnostic laparoscopy in all women with unexplained infertility to identify and treat those with early-stage endometriosis is questionable since the absolute increase in pregnancy is modest (3, 7). For more advanced stages, surgery may represent a treatment option as it may improve their fertility prognosis (3).

More evidence exists about the usefulness of operative laparoscopy for the treatment of endometrioma-associated infertility as it may increase the chance of natural pregnancy (8).

However, some researchers have expressed concern regarding the negative effect of the traditional surgery (cystectomy) on ovarian reserve by way of removing healthy ovarian tissue and vascular injury (9). When performing a cystectomy, the experience of the surgeon could be very important in relation to ovarian damage. Cystectomy is not an easy procedure and when performed by an inexperienced surgeon, increased damage to healthy ovarian tissue may occur (10). For these reasons, some authors suggested that ablative techniques may represent a less aggressive approach toward the healthy ovarian cortex, as long as the energy employed avoids thermal diffusion to the surrounding ovarian tissue (11, 12).

In our daily practice, we have introduced ablation using CO2 fiber laser technology: it is simple, easy to use, and has the advantage of eliminating the “surgeon's experience” factor (13). We have already been able to report encouraging results in terms of ovarian reserve preservation, postoperative pregnancy rates, recurrence risk and ovarian responsiveness in ART (14–16).

Another ablative technique is sclerotherapy, which consists of injecting a sclerosing agent into the cyst cavity, destroying endometrial tissue inside the cyst while sparing a woman's ovarian reserve. However, this procedure does not allow to have a histological diagnosis of endometriosis and can cause abdominal pain, abscess formation, infection, thus limiting its use in clinical practice (17).

Therefore, the aim of this paper is to review the impact of the two surgical technique for ovarian endometriosis treatment of cystectomy and CO2 fiber laser vaporization focusing on reproductive outcomes. We included only studies involving humans and published in the English language.

### 2.1. Cystectomy

Laparoscopic cystectomy represents the reference standard for surgical treatment for ovarian endometriosis according to the latest Cochrane review published in 2008 (18). Cystectomy consists in stripping away the cyst wall from the underlying healthy ovarian parenchyma by delicate traction and countertraction maneuvers followed by selective hemostasis. However, endometrioma is a “pseudocyst” with no clear cleavage plan, thus the risk of inadvertent removal of healthy ovarian parenchyma is higher compared to other ovarian benign cysts especially in case of surgeons without an extensive expertise in the endometriosis surgical treatment (6, 19, 20). The body of literature published during the past decades regarding the effect of surgery on ovarian reserve markers and either spontaneous or IVF/ICSI pregnancy rate is consistent. However, the heterogeneity in cysts size, surgical technique, number of endometriomas surgical treated, IVF/ICSI protocols and AMH/AFC assessment, does not allow to draw definitive conclusions on the amount of ovarian damage following cystectomy.

#### 2.1.1. Ovarian reserve markers following cystectomy

Endometrioma cystectomy, even when performed by an expert surgeon, may lead to significant ovarian tissue removal which increases proportionally as cyst diameter increases (19). That may result in diminished ovarian function. The effect of cystectomy on ovarian reserve markers in terms of antimullerian hormone (AMH) has been extensively addressed by recent systematic review and meta-analysis by Younis et al. (21) which included only prospective studies (22, 23). The pooled analysis revealed a decrease in AMH value in the short term followed by a slight improvement during the intermediate period (21). AMH reduction was more pronounced for bilateral cystectomy compared to unilateral with 53.9% vs. 38.4% decrease for the very short term period (1 week to 1 month) and 43.4% vs. 26.9% for the intermediate period (6 weeks to 6 months). This result may suggest that a certain amount of ovarian reserve damage might be reversed. However, the partial ovarian recovery at 6 months after surgery may be temporary since AMH post-operative decrease in the long term (9–12 months) is even worse than 1 month after surgery with 57% and 39.5% reduction for bilateral and unilateral cystectomy from the baseline (21).

Many other previous retrospective studies warned about the risk of postsurgical ovarian failure after cystectomy of bilateral endometriomas which ranged between 1.7% (9) and 2.4% (24) even by anticipating the age of menopause (25, 26). Moreover, Benaglia et al. observed absence of follicular growth in around the 13% of operated ovaries.

#### 2.1.2. Spontaneous pregnancy outcomes following cystectomy

Very few studies have investigated the rate of spontaneous pregnancy rate between women who underwent cystectomy and those with endometriomas in place. Most of them aimed to investigate only spontaneous pregnancy rate after cystectomy with plenty of multiple confounding factors, including selection bias (unilateral and bilateral endometriomas, different cyst size). Moreover, when a unilateral endometrioma was present, the operated gonad could have been damaged and pregnancy achieved as a result of the function of contra-lateral adnexa.

In a meta-analysis by Vercellini et al. (8), the chance of pregnancy after laparoscopic excision of endometriomas ranged from 30% (27) to 67% (28) according to ASRM stage. However, all the stages had similar percentages at 36° month (29).

Dubinskaya et al. (30) investigated the spontaneous pregnancy rate after unilateral cystectomy dividing women into 2 groups according to AMH levels (< or >2 ng/ml) before surgery. Cumulative pregnancy rate in the following year was 30.3% for women with AMH <2 ng/ml vs. 54.7% for those with AMH >2 ng/ml. The majority of patients in both groups became pregnant in the first 6 months compared to the second half of the year after surgery. These conclusions are consistent with the results of Zhu, Pantou et al. and Taniguchi et al. When the likelihood of spontaneous pregnancy after laparoscopic cystectomy of endometriomas was compared with other benign ovarian cysts, it was observed that it is more than 3 times higher in the group of patients with other benign tumors compared to endometriomas (HR 3.57; *p* = 0.03) (31). Thus, it seems that the best outcomes in terms of pregnancy performance are achieved in the very first period after surgery, that this beneficial effect is more pronounced for stage III and tends to disappear as the time pass, that the preoperative AMH has a predictive value for pregnancy and that endometriosis per sè may affect the chance of pregnancy. However, it must be considered that the presence of adenomyosis or a high ASRM total score where a complete resection of endometriosis could not be achieved, may reduce pregnancy rate (32, 33). Thus, these factors should be prompt recognize to further guide management decisions for individualization of care.

#### 2.1.3. IVF outcomes following cystectomy

The impact of unilateral cystectomy vs. intact endometrioma was analyzed by Hamdan et al. (34). No differences were found in terms of LBR, CPR, MNOR and cancellation rate per cycle even though women with endometrioma surgically treated required a higher dose of FSH during controlled ovarian stimulation. However, some previous studies underlined that bilateral endometriotic cystectomy is characterized by a high rate of IVF failure (no embryos to be transferred obtained) and reduced live birth rate (35, 36). Interestingly, according to Roustan et al. (37) it seems that previous cystectomy might lead to an even worse ovarian response to IVF/ICSI protocols in terms of implantation, pregnancy and live birth rates per cycles when compared to that of women with diminished ovarian reserve markers but no previous ovarian surgery. Although the authors did not distinguish between uni- or bilateral cystectomy, it could be postulated that the magnitude of the detrimental effect of cystectomy on IVF/ICSI outcomes would be worse in case of bilateral endometrioma(s).

### 2.2. CO2 fiber laser ablation

The fear of ovarian failure following cystectomy has driven clinicians to perform ablative techniques. In this surgical approach, the “pseudo-capsule” is not removed but it is ablated with energies with little thermal spread. Among the several sources of energy investigated so far, CO2 fiber laser has showed promising results in the treatment of endometrioma-associated infertility. After its validation by Kaplan and his colleagues in 1973, from the early 1980′s to 1990′s Nezhat brothers et al. had optimized the use of CO2 laser for laparoscopic treatment of endometriosis (38, 39). CO2 lasers emit light at a wavelength of 10,600 nm that is absorbed strongly by water: radiant energy is converted to heat, instantly raising the temperature of tissue water to more than 100°C and thus vaporizing the target lesion (40). Compared with all available energy sources, CO2 laser is highly selective and precise, has minimal depth of tissue penetration (41) and produces little lateral thermal spread, lowering the risk of unintended thermal damage produced by non-visible larger necrosis and/or deep penetration (42). Furthermore, being used in a non-contact mode, CO2 laser allows continuous visualization of the section plane between healthy and endometriosis affected tissue. These properties are of crucial importance when attempting to preserve the surrounding viable ovarian tissue. In addition, CO2 laser simultaneously cauterizes bleeding tissue making hemostasis very effective without the risks of cautery (43). Historically CO2 lasers used to be fixed to rigid instruments lending to ergonomic difficulties, however newer technologies have allowed for a flexible fiber delivery system, overcoming past ergonomic challenges by providing flexibility, durability, and ease of use. However, CO2 laser may have some disadvantages. This non-excisional technique may be associated with higher recurrence rates (18), possibly as a consequence of the less aggressive nature of the surgery. Furthermore, to confirm the diagnosis of endometriosis during CO2 laser ablation, only a small portion of the endometrioma's pseudocapsule is biopsied, in order to respect as much as possible, the ovarian tissue. Nevertheless, considering endometriosis a benign condition, which affect young women of reproductive age, the risk of a lack of diagnosis for malignant condition is unlikely, especially because women are carefully evaluate through a presurgical transvaginal ultrasound scan to detect any sign of suspicious lesion before surgery.

In conclusion, due to its intrinsic properties of ovary sparing surgery, endometrioma treatment using CO2 fiber laser ablation has showed several benefits in terms of post-operative reproductive outcomes.

#### 2.2.1. Ovarian reserve following CO2 fiber laser ablation

There are consistent data in the literature about the safety and efficacy of CO2 laser technology. Recently, our group reported a significant improvement in the AFC of the operated ovary and no change in AMH levels after CO2 fiber laser vaporization compared to cystectomy in unilateral endometrioma (11). Moreover, in patients randomized to laser vaporization, we demonstrated similar ovarian volumes between the operated ovary and the contralateral non-operated ovary (11). These findings are consistent with those reported by Tsolakidis et al. (44).

Only few studies have been published so far regarding the impact of surgery on ovarian reserve in bilaterally treated endometriomas. A randomized trial including patients with bilateral endometriomas was conducted by Rius et al. (45) reporting that mean ovarian volume and AFC after surgery were significantly higher in the laser-treated ovaries compared to those treated by stripping.

Taken together, these findings suggest a lower impact on ovarian reserve after CO2 laser ablation rather than after the stripping procedure.

#### 2.2.2. Spontaneous pregnancy outcomes following CO2 fiber laser ablation

Due to the recent introduction of laser CO2 in clinical setting, very limited data so far have been published regarding postoperative pregnancy rates after this procedure. Remarkably, the few studies available are based on retrospective design with convenient sampling of enrolled population. Therefore, larger and unbiased studies, with longer follow-ups are needed to assess the chance of spontaneous pregnancy after endometrioma ablation throughout CO2 laser. Our group recently reported comparable probabilities of postoperative spontaneous pregnancy in patients treated with CO2 laser vaporization or cystectomy (35.9% vs. 55.6% respectively, *p* = NS) (11). Similar data were reported by Carmona and collegues at 12 months follow-up after cystectomy or vaporization (46) and also for bilateral endometriomas, with 33% of pregnancy reported at 6 month follow-up (45).

#### 2.2.3. IVF outcomes following CO_2_ fiber laser ablation

With regards to the ART setting, Donnez et al. found similar IVF outcomes in patients treated with CO2 laser vaporization when compared to controls without endometriosis (47, 48). These positive findings are consistent with those observed in our prospective observational study investigating ovarian responsiveness to controlled ovarian stimulation (COS) after CO2 laser vaporization (49). Moreover, when comparing IVF outcomes in patients carrying small (<3 cm) endometriomas at the time of ART and women treated with CO2 fiber laser vaporization before ART, no significant differences were found between the two groups (16). Remarkably, a greater number of good-quality embryos were observed in both surgical groups, suggesting a potential benefit of surgery in reducing local inflammation and oxidative stress, perhaps restoring a functional ovarian microenvironment. Finally, the cumulative pregnancy rate of the two groups were comparable (38.9% in CO2 fiber laser group vs. 40% in patients carrying endometrioma; *p* = NS).

These results suggest that CO2 laser-treated endometrioma is associated with favorable fertility outcomes.

## 3. Further perspectives: ovarian microvascular flow following CO2 fiber laser ablation

During the last decades there has been a major push by researchers into finding and investigating new techniques to reach the patients individual treatment goals with the least adverse effect on the healthy ovarian tissue. Most of current knowledge regarding post-operative follow-ups is based on common ovarian reserve parameters and pregnancy outcomes; no investigation regarding more precise parameters such as microvascular ovarian flow after surgery has been accomplished so far. This is quite remarkable, considering that there are evidence attributing to laser therapy also some healing properties (50). Indeed, it has been suggested that laser treatment might stimulates low-level mitochondrial generation of free radicals and hydrogen peroxide which are known to play a role in intracellular signaling by triggering intracellular calcium mobilization. In particular, free radicals have shown ability to activate certain transcription factors which can stimulate gene expression of several proteins including also Vascular Endothelial Growth Factor (VEGF), a key protein promoting neo-angiogenesis (51).

Believing in such healing properties and consequently in a possible role of this technique in restoring ovarian reserve, few months ago our group has implemented a highly specific and specialized prospective protocol aiming to evaluate ovarian volume and microvascular flow after treatment. Using the newly introduced SAMSUNG HERA w10 Ultrasound (US) machine, beyond classical morphological evaluation, we perform a structured evaluation of color and pulsed-waved doppler as well as three-dimensional structural analysis of the ovaries treated combined with power doppler (PW). The aim of this prospective observational study is to try to understand the physiology behind increased ovarian functionality after doing CO2 laser therapy when compared to cystectomy. The study was approved by the hospital's Ethics Committee for compassionate purposes. In the study protocol, patients treated by endometrioma CO2 fiber laser vaporization undergo this specialized TVUS evaluation on two occasions to evaluate the changes in time, the first at one month from the surgery and the second at three months from the surgery. The following microvasculature-related parameters are collected: end diastolic velocity (EDV), end systolic velocity (ESV), resistance index (RI), pulsatility index (PI), vascularity index (VI), flow index (FI) and vascularity-flow index (VFI). At the moment, owing to the recent implementation of this study protocol, prospective data of only 10 patients treated with CO2 fiber laser vaporization have been fully collected. After surgery, no statistically significant differences in terms of microvasculature-related parameters were found between the operated ovary and the contralateral non-operated ovary. Interestingly, an overall trend towards an improved microvascular flow after surgery is observed. We believe that a larger sample size and a longer follow-up might will help in reaching the statistical power needed to strengthen the reliability of these preliminary findings, therefore confirming the benefits of CO2 fiber laser vaporization also on microvascular flow of treated ovaries.

## 4. Conclusions

Minimally invasive surgery plays a decisive role in many clinical situations associated with infertility. More specifically, clinicians may consider operative laparoscopy for the treatment of endometrioma-associated infertility as it may improve reproductive outcomes. In the field of endometriosis management, new technology has been developed in the last decades. Although there is little data available so far, CO2 fiber laser vaporization appears to be a promising method to treat endometriomas, to preserve ovarian function and to improve fertility prognosis in infertile women.

## Author contributions

All authors contributed to the article and approved the submitted version.
